# Left ventricular ejection fraction by real-time three-dimensional echocardiography: The Necker cube for the naive realism of two-dimensional methods

**DOI:** 10.1007/s12471-014-0579-z

**Published:** 2014-08-12

**Authors:** B. M. van Dalen

**Affiliations:** 1 Department of Cardiology, The Thoraxcenter, Erasmus University Medical Center, Rotterdam, the Netherlands; 2 Department of Cardiology, Sint Franciscus Gasthuis, Kleiweg 500, 3045 PM Rotterdam, the Netherlands; 3 Erasmus MC, Het Thoraxcentrum, Polikliniek Cardiologie, ‘s-Gravendijkwal 230, 3015 CE Rotterdam, the Netherlands

Despite a staggering technical progress in echocardiography, in daily clinical practice assessment of left ventricular (LV) ejection fraction (EF) is still often done in a rather inaccurate two-dimensional (2D) manner. Real-time three-dimensional echocardiography (RT3DE) provides a more *representative realistic* view of LV volumes, thereby being the Necker cube for the *naive realism* of 2D methods.

The Necker cube (Fig. 1a) is an optical illusion first published by the Swiss crystallographer Louis Albert Necker in 1832 [1]. It provides a counter-attack against *naive realism,* which states that the way we observe the world is the way the world actually is. The Necker cube contradicts this claim, because we see one or the other of two cubes (Fig. 1b), but really, there is no cube there at all, but only a 2D drawing of 12 lines. We see something which is not really there, thus apparently disproving *naive realism*. This criticism of *naive realism* supports *representative realism*. This view argues that we experience reality indirectly by perceptions that represent the real world. For example, if we see a yellow flower, we do not actually see the flower itself but a representation of it. In this way, differences of perception which occur due to changes in the position of the viewer, light conditions, and so on can be easily explained: it is not the object that is changing, only our perception of it.

**Fig. 1:**
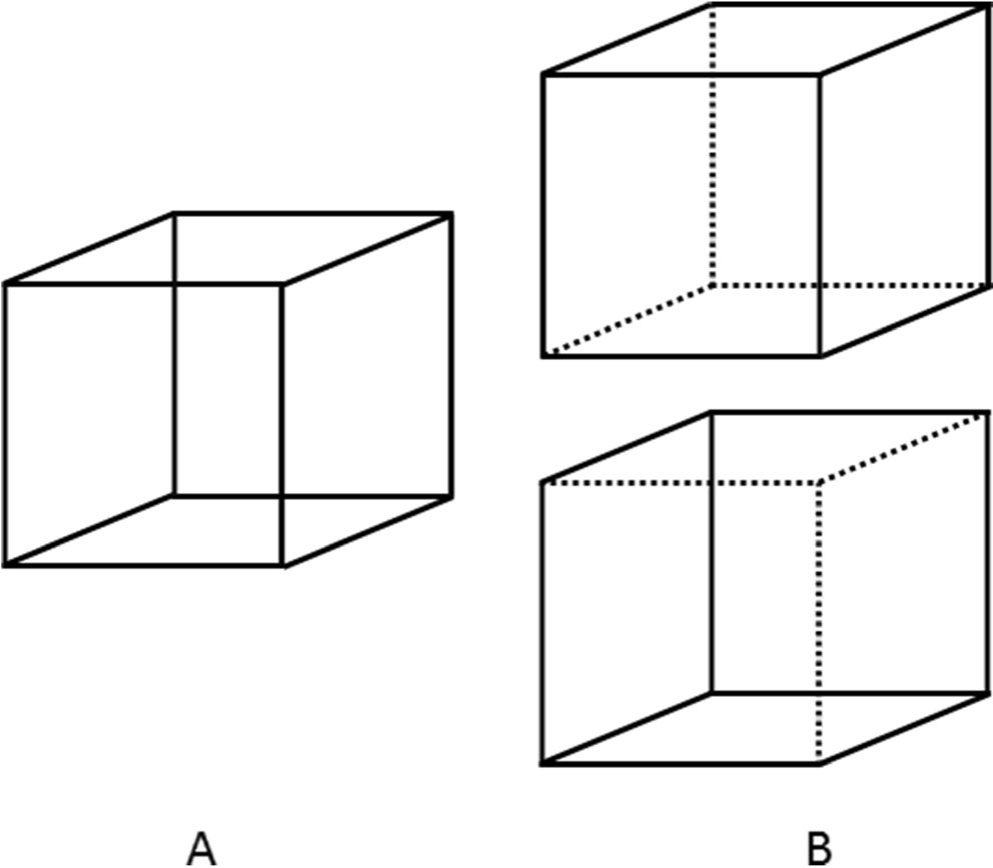
**a** The Necker cube. **b** Like a 2D echocardiographic image, the Necker cube may give the suggestion of a realistic representation of a 3D structure, but really there is only a 2D picture that can be interpreted in different ways

Let us go back to echocardiography. Visual estimation of wall motion (‘eyeballing’) is highly subjective, imprecise and poorly reproducible. Nevertheless, some sense of *naive realism* seems to provide an unrealistic confidence in the accuracy of our eyes. Calculating LV volumes and EF from 2D images by using algorithms such as the biplane method of disks (modified Simpson’s rule) are considered more accurate and better reproducible. However, because of the geometric assumptions of this 2D method, measurements may be inaccurate if the shape of the left ventricle is abnormal or when the acquisition of the 2D images is suboptimal. In other words, like the yellow flower, 2D echocardiographic images are not necessarily a realistic representation of the true left ventricle, but only a perception that is influenced by subjective interpretation and variability in measurements of only a very small 2D portion of the true 3D volume.

## Left ventricular volumes and ejection fraction by real-time 3D echocardiography

The advent of matrix transducers, together with impressive improvements in semi-automated volumetric analysis, have allowed 3D echocardiography to evolve from a complicated and time-consuming research tool into a simple and fast imaging modality ready for everyday clinical use. RT3DE has been extensively demonstrated to be more time-saving, reproducible and accurate than conventional 2D echocardiography [2]. One of the important factors that may have led to delayed acceptance of RT3DE in daily clinical practice may be the intervendor inconsistency of 3D quantitative parameters. Therefore, Driessen et al. [3] should be congratulated on their effort to compare two of the big players in this field.

Although contemporary RT3DE analysis software allows assessment of LV volumes and EF in a rather fast and simple way, a certain learning curve still needs to be taken into account. Driessen et al. found that RT3DE software was most accurate in the hands of more experienced observers. Observers were regarded more experienced when they had been working on RT3DE analyses for more than 3 months. Noteworthy, even after 3 months of working on RT3DE, increasing experience may still lead to better results, as shown in one of the landmark papers with respect to quantification of LV volumes by RT3DE [4]. The level of experience in this study ranged from several hours of instruction to at least 1 year of frequent use. Measurements performed by the most experienced investigators showed differences with cardiac magnetic resonance imaging (CMR) that were roughly half of those noted in the entire study group.

## Is magnetic resonance imaging the perfect reference technique?

RT3DE provides LV volumes smaller than those derived from CMR. CMR is considered the reference technique for assessment of ventricular volumes. However, there are intrinsic limitations of CMR as a reference technique because of its multislice rather than true 3D nature.

Usually, as in the study by Driessen et al., CMR-derived volumes are calculated by a disc summation technique (in other words, adding the volumes of several, usually 8 mm thick, slices). Yet, with in vitro measurements, it was shown that this disc summation may result in an overestimation of 20 % of the true volume. A volumetric 3D CMR analysis resulted in more accurate measurements and RT3DE only slightly underestimated true volumes [5].

The differences between LV volume analysis by RT3DE and CMR have also been attributed to the sometimes insufficient image quality of RT3DE. Blurring of the endocardium may cause difficulty in clearly identifying the endocardial-trabecular border. As a result, with manual tracing of RT3DE images, the human eye tends to trace the inner border of the blurred rim of the left ventricle, which may be the blood-trabecular interface rather than the true endocardial surface. On the other hand, partial volume effects caused by the relatively thick slices in CMR result in the appearance of a fairly smooth surface of the endocardium, which may also be a false representation of the truth.

Nevertheless, both the direct 3D volumetric echocardiographic method by Philips and the 3D speckle tracking echocardiographic method by Toshiba, used in the study by Driessen et al., use automated systems to detect the endocardium. This line is supposed to be closer to the centre of the blurred area and these mathematical methods may happen to include more cavity than a manual tracking method, resulting in less underestimation.

## Future directions

There is room for improvement. Accurate LV quantification by RT3DE can only be performed using good image quality datasets, usually obtained in 80–85 % of routine patients, a number also found in the ‘daily clinical practice study’ by Driessen et al. Also, regular heart rhythm (Driessen et al. excluded patients with arrhythmia) and patient cooperation for breath holding are still indispensable. And finally, the relatively low temporal resolution of RT3DE limits the assessment of regional wall motion during exercise and dobutamine stress echocardiography [2]. Nevertheless, as mentioned before, contemporary RT3DE does beat 2D echocardiography in terms of reproducibility and accuracy. It seems to provide a Necker cube-like counter-attack against the *naive realism* of believing everything we measure using 2D echocardiographic images. A sense of *representative realism* seems to be a welcome alternative to the *naive realism* of confidence in the accuracy of our eyes and 2D measurements. In my opinion, especially in case of good image quality, RT3DE should be the preferred method for measurement of LV volumes and EF.
